# Genetic Variants Associated with Response to GLP-1 Receptor Agonists in Diabetes and Obesity

**DOI:** 10.3390/life16091486

**Published:** 2026-09-05

**Authors:** Mónica T. Fernandes, Joana C. Dias, Margarida Espírito-Santo, Maria Dulce Estêvão, Ana Luísa De Sousa-Coelho

**Affiliations:** 1 Faculdade de Medicina e Ciências Biomédicas (FMCB), Universidade do Algarve, 8005-139 Faro, Portugal; mafernandes@ualg.pt; 2 Algarve Biomedical Center Research Institute (ABC-Ri), Universidade do Algarve, 8005-139 Faro, Portugal; 3 Faculdade de Ciências e Tecnologia (FCT), Universidade do Algarve, 8005-139 Faro, Portugal; 4 Escola Superior de Saúde, Universidade do Algarve (ESSUAlg), 8005-139 Faro, Portugal

**Keywords:** diabetes, genetic variants, glucagon like peptide 1 (GLP-1, GLP1) analogs, GLP-1 receptor (GLP-1R, GLP1R), GLP-1 receptor agonist (GLP-1RA), incretins, obesity, polymorphisms

## Abstract

Genetic variation may contribute to interindividual differences in the efficacy and tolerability of glucagon-like peptide-1 receptor agonists (GLP-1RAs), although the available evidence remains limited. Certain gene variants may underlie variability in glycemic control and weight loss outcomes among patients treated with GLP-1RAs for diabetes and/or obesity. A better understanding of these genetic variations could have significant implications for the development of personalized medicine, contributing to predicting patient responses to GLP-1RAs. Within this review, we collected and synthesized information from different studies about genetic variants that have been reported to be associated with altered therapeutic response to GLP-1RAs, mostly in *GLP1R*, but also in *TCF7L2* and *PNPLA3* genes. For the same variants, the differences obtained in the outcomes were, however, inconsistent between studies. Such disparities may partly reflect the diversity of the outcomes analyzed, the specific GLP-1RA in use, and/or the characteristics of each population. Additional genes, such as *PPARD*, *WFS1* and *VTRNA2-1*, exhibited differences in at least one study. Although still limited, considering the fast expansion of the prescription of this therapeutic class, these results reflect the need for additional studies, before enabling the implementation of pharmacogenetics in clinical practice.

## 1. Introduction

Diabetes mellitus (DM) is a cause for concern all over the world and is a huge public health challenge [1]. It is characterized by insulin resistance and the consequent progressive loss of adequate insulin secretion by pancreatic β-cells, resulting in a state of chronic hyperglycemia [2]. Risk factors such as genetics, age, obesity (especially abdominal or visceral), a sedentary lifestyle and the existence of other comorbidities such as hypertension and dyslipidemia can contribute to the development of type 2 diabetes (T2DM) [3]. The goals of the treatment for T2DM are to prevent or delay the development of complications and to optimize the quality of life of individuals living with the disease. Glucose-lowering drugs currently recommended to treat this disease belong to the following pharmacotherapeutic groups: biguanides (metformin), sulfonylureas, thiazolidinediones (pioglitazone), dipeptidyl peptidase-4 (DPP-4) inhibitors, sodium-glucose co-transporter 2 inhibitors (SGLT2, sodium-glucose co-transporter-2), glucagon-like peptide-1 (GLP-1) receptor agonists (GLP-1RAs), dual (glucose-dependent insulinotropic polypeptide) GIP/GLP-1RA (tirzepatide), and insulin [4]. Current GLP-1RAs in clinical practice include semaglutide, liraglutide, exenatide, dulaglutide, and lixisenatide. In addition to their glucose-lowering effects, some GLP-1RAs, including semaglutide and liraglutide, are approved for chronic weight management in individuals with obesity or overweight with weight-related comorbidities, including those without T2DM. Tirzepatide is also approved for weight management in these populations [5].

Pharmacogenetics studies the impact of individual genetic differences on the action, use and dosage of a drug. The effectiveness of a particular drug can be altered by the existence of genetic variants that lead to changes in the pharmacodynamics or pharmacokinetics of the drug, namely changes in its transport or metabolism [6]. Genetic variants include single nucleotide variants, of which common variants are referred to as single-nucleotide polymorphisms (SNPs), as well as structural variations such as deletions, insertions, inversions or copy number variants [7,8]. In clinical practice, the choice of treatment for T2DM (and for most of the diseases, including obesity) is often based on a trial-and-error process according to a set of factors that does not consider the genotype of the individuals, highlighting the need for more robust knowledge about genetic variants that may pave the way to precision medicine in these diseases [9,10].

Considering that genetic variants can contribute to altering the pharmacokinetics or pharmacodynamics of a drug, gathering not only clinical and physiological information, but also selected genetic information, can be an asset for clinical decision-making, since it may allow predicting who is most likely to benefit from a given treatment. Based on the substantial increase in the prescription and use of GLP-1RAs over the last years (including expanding indications) [11,12,13], the main goal of this review was to collect and synthesize information on the existence of genetic variants that were associated with altered therapeutic response to GLP-1RAs in the treatment and management of T2DM and obesity.

## 2. Materials and Methods

### 2.1. Type of Study and Search Strategy

This review was conducted as a critical narrative synthesis of the available evidence using a systematized methodology. A structured search strategy, predefined eligibility criteria, independent screening, and standardized data extraction procedures were employed to maximize the identification of relevant evidence. Quality appraisal of the studies was not performed. An advanced search was carried out on 24 April 2025, in PubMed, Scopus and Web of Science, using the combination of search terms shown in Table S1. No time or other restrictions were applied. During manuscript revision, a supplementary search was conducted. The studies identified were assessed according to the original eligibility criteria and, when relevant, were incorporated into the Discussion to contextualize recent developments. However, they were not included in the formal evidence synthesis, data extraction process, or summary tables. Although systematic methods were applied, this review does not constitute a formal systematic review.

### 2.2. Inclusion and Exclusion Criteria

Original articles were included if studied individuals were being treated for T2DM and/or obesity with GLP-1RAs, and there was an evaluation of the existence of genetic variants that could lead to changes in the therapeutic response in terms of glycemic control or weight loss. Only human studies, including either randomized clinical trials or observational studies, were selected. Articles reporting exclusively animal or *in vitro* studies, reviews, meta-analyses and cross-sectional studies were excluded.

### 2.3. Screening and Data Extraction

Titles and abstracts were initially analyzed independently by two researchers, using the Rayyan platform [14], to identify articles that potentially met the inclusion criteria. The full text of these studies was then consulted to assess their eligibility. The following information was extracted from each study: title, authors, year of publication, country, size and characteristics of the population, gene and genetic variant studied, drug used, and therapeutic response identified. Additional variables, namely sex, age, body mass index (BMI) and glycated hemoglobin (HbA1c), were also collected, whenever available. When studies reported additional outcomes, namely hepatic parameters, markers of β-cell function, gastric emptying, methylation profiles, or adverse effects, these data were also collected and narratively synthesized as secondary or exploratory outcomes. No statistical methods were used.

## 3. Results

### 3.1. Characteristics of Included Studies

A total of 21 studies investigating the influence of genetic variants on therapeutic response to glucagon-like peptide-1 receptor agonists (GLP-1RAs) were included in this review (Table 1). The studies were published between 2013 and 2024 and comprised a heterogeneous body of evidence, including genome-wide association studies, pharmacogenetic cohort studies, retrospective observational analyses, and post hoc analyses of clinical or interventional studies. Collectively, the studies originated from diverse geographic regions, including Europe, Asia, North and South America, and the Middle East, with one additional multinational study.

Sample sizes varied considerably, ranging from small pharmacogenetic cohorts to a large multicenter analysis involving more than 4500 individuals with T2DM. Most studies enrolled adults with inadequately controlled T2DM (with or without obesity, including other morbidities such as hepatic steatosis or polycystic ovary syndrome (PCOS)), although some also included participants with obesity without a mandatory diagnosis of T2DM (Table S2). Baseline metabolic characteristics were similarly heterogeneous, with mean BMI values generally falling within the overweight or obesity range, and baseline HbA1c values typically reflecting suboptimal glycemic control before GLP-1RA initiation.

The GLP-1RAs evaluated included liraglutide, exenatide, dulaglutide, semaglutide, lixisenatide, and albiglutide, with liraglutide and exenatide being the most frequently investigated agents. In several studies, GLP-1RAs were administered as add-on therapy to standard glucose-lowering regimens, particularly metformin, sulfonylureas, sodium-glucose cotransporter-2 inhibitors, or insulin-based therapies. Mean treatment duration ranged from 1 month to over 1 year (Table 1), depending on the study design and evaluated outcomes (Table S2).

Across the included studies, genetic variants were evaluated in relation to several measures of therapeutic response, most commonly glycemic outcomes, including reductions in HbA1c, fasting plasma glucose, postprandial glycemia, insulin secretion, and indices of β-cell function. Weight-related outcomes, including changes in body weight, BMI, waist circumference, or percentage weight loss, were also assessed in a subset of studies. By contrast, adverse effects and treatment tolerability were less frequently evaluated as potential pharmacogenetic-related outcomes.

### 3.2. Genetic Variants Associated with GLP-1 Receptor Agonist Response

#### 3.2.1. Stratified Analysis by Genotype Studies

Studies using genotype-based stratification constituted a substantial proportion of the evidence included in this review. These studies assessed whether predefined genetic variants were associated with differences in glycemic control, weight-related outcomes, insulin secretion, β-cell function, hepatic parameters, or composite definitions of therapeutic response following treatment with GLP-1RAs. The most frequently investigated genes were *GLP1R*, which encodes the pharmacological target of the GLP-1RAs, and genes involved in glucose metabolism, β-cell function, obesity susceptibility, hepatic metabolism, or cardiometabolic regulation, including *TCF7L2*, *KCNQ1*, *WFS1*, *PNPLA3*, *PPARD*, *CNR1*, and *VTRNA2-1*. Additional gene variants studied were in *CTRB1/2*, *CHST3*, *TMEM114*, *CETP* and *IL6R* genes (Table 2).

***GLP1R* variants**

Variants in *GLP1R* were the most frequently investigated genetic variants in studies evaluating outcomes during GLP-1RA treatment. Across the included studies, the main *GLP1R* single nucleotide variants investigated were rs10305420 [23,29,31], rs6923761 [22,30,31,33], and rs3765467 [23,29]. Additional variants, including rs1042044, were evaluated in individual studies, sometimes alongside variants in other genes related to incretin signaling or metabolic response [31], as described below.

The *GLP1R* rs10305420 C>T variant was assessed in several studies, but with heterogeneous findings. In a 12-week study evaluating exenatide or liraglutide in combination with metformin or other glucose-lowering drugs, Guan et al. reported no significant differences between rs10305420 genotypes in body weight or glycemic control, including HbA1c [23]. By contrast, Yu et al., in a study with exenatide combined with metformin for 6 months, found that the variant allele was associated with a decreased effect in the reduction in both body weight and HbA1c [29]. In adults without T2DM treated with exenatide combined with dapagliflozin for 24 weeks, Pereira et al. found no significant association between rs10305420 and body weight change [31].

The *GLP1R* rs6923761 G>A variant was mainly evaluated in relation to weight-related outcomes. In a 14-week liraglutide study in patients treated with metformin, de Luis et al. reported significant reductions in BMI, body weight, and fat mass in both GG homozygotes and A-allele carriers. However, larger decreases in BMI, body weight, and fat mass were observed among carriers of the variant allele. In contrast, reductions in basal glucose, homeostatic model assessment of insulin resistance (HOMA-IR), and HbA1c were similar between genotype groups, suggesting that this variant may be more strongly related to anthropometric response than to glycemic response in that cohort [33]. Maselli et al. did not identify statistically significant differences between rs6923761 genotype groups after liraglutide treatment [22]. Similarly, Chedid et al. reported a non-significant difference in weight loss according to rs6923761 genotype after 4–5 weeks of GLP-1RA therapy with exenatide or liraglutide [30]. The results of Pereira et al. for the same variant also showed no significant differences between genotypes [31].

The *GLP1R* rs3765467 G>A variant was evaluated in relation to both glycemic and weight-related outcomes. Guan et al. found no significant differences in body weight according to rs3765467 genotype after 12 weeks of exenatide or liraglutide therapy. However, HbA1c reduction was significantly greater in individuals carrying the GG genotype compared with carriers of the GA or AA genotypes [23]. By contrast, Yu et al. found no significant association between rs3765467 and either body weight reduction or HbA1c reduction after exenatide plus metformin treatment [29].

***TCF7L2* and other β-cell function-related variants**

The *TCF7L2* rs7903146 C>T variant was another frequently assessed variant. Maselli et al. reported that individuals with the CC genotype achieved lower body weight at 16 weeks after liraglutide treatment compared with carriers of the CT or TT genotypes, while similar at baseline [22]. However, Chedid et al. found no significant association between rs7903146 genotype and weight change after treatment with exenatide or liraglutide [30]. Ferreira et al. evaluated the impact of *TCF7L2* rs7903146 on metabolic response to exenatide over 8 weeks. No significant differences were observed between genotype groups in body weight reduction or fasting plasma glucose (FPG) levels after treatment [28].

Other variants related to β-cell function or glucose metabolism were evaluated less frequently. Pereira et al. assessed *TCF7L2* rs7903146, *KCNQ1* rs151290 and *WFS1* rs10010131 in adults without diabetes treated with exenatide and dapagliflozin. Among these, only *WFS1* rs10010131 was significantly associated with body weight change, with greater weight loss observed in carriers of the A/G or A/A genotypes compared with GG homozygotes [31].

The *KCNQ1* gene was evaluated in an additional study using a different genetic variant. Geng et al. analyzed *KCNQ1* rs163184 T>G and found that carriers of the TG or GG genotypes had a worst glycemic response to 12 months of exenatide therapy, reflected by smaller reductions in HbA1c and FPG [24]. In contrast, Pereira et al. found no significant association between *KCNQ1* rs151290 and body weight change in adults treated with exenatide and dapagliflozin [31].

***PNPLA3* variants**

The *PNPLA3* rs738409 C>G variant was evaluated in studies assessing metabolic and hepatic outcomes after GLP-1RA treatment. Gavriilidis et al. reported that *PNPLA3* rs738409 was associated with the effect of dulaglutide on HbA1c after 6 months of treatment, although hepatic scores did not show differences [15].

Urias et al. evaluated *PNPLA3* rs738409 in patients treated with semaglutide and assessed outcomes at 6, 12, and 24 months. BMI changes were similar across *PNPLA3* genotype groups, suggesting no clear genotype association to weight reduction in that cohort [17]. However, in this study, hepatic outcomes appeared to differ according to genotype, with alanine aminotransferase (ALT) levels decreasing more markedly in carriers of at least one risk allele after one year of semaglutide therapy [17].

Finally, Chen et al. assessed *PNPLA3* rs738409 in patients treated with exenatide for 6 months. In this study, exenatide treatment did not lead to statistically significant reductions in BMI or HbA1c in either genotype group [27].

***PPARD* variants**

Song et al. investigated the association of *PPARD* rs2016520 T>C and *PPARD* rs3777744 A>G with response to 6 months of exenatide monotherapy. The *PPARD* rs3777744 variant was associated with several metabolic response parameters. Individuals with the AA genotype showed greater reductions in HbA1c and HOMA-IR, whereas carriers of the AG or GG genotypes had attenuated responses with respect to HbA1c, waist-to-hip ratio, FPG, and HOMA-IR. No significant association of rs3777744 variants and BMI response was observed [21].

Regarding *PPARD* rs2016520, no significant association was observed with HbA1c response. However, carriers of the C allele showed greater improvement in HOMA-IR compared with individuals with the TT genotype, suggesting a possible influence on insulin resistance-related outcomes. As with rs3777744, rs2016520 was not significantly associated with BMI change after exenatide treatment [21].

***CNR1* variants**

The *CNR1* rs1049353 G>A variant was evaluated by de Luis et al. in patients with obesity and type 2 diabetes treated with liraglutide in combination with metformin, with or without sulfonylureas. In both genotype groups, including GG homozygotes and A-allele carriers, liraglutide treatment was associated with reductions in BMI, body weight, fat mass, waist circumference, and systolic blood pressure. However, the magnitude of these reductions was similar between genotype groups [34].

***VTRNA2-1* genetic and epigenetic variation**

Lin et al. evaluated the *VTRNA2-1* rs2346018 A>C variant and also the status of *VTRNA2-1* promoter methylation in relation to glycemic response to GLP-1RA therapy. The variant was associated with methylation density but was not independently associated with glycemic response. A stronger association with poor glycemic response was observed when carriage of the rs2346018 A allele was combined with promoter hypomethylation [26]. This finding should therefore be interpreted as a combined genetic–epigenetic association rather than as an independent genotype effect.

**Other candidate variants**

Additional candidate variants were assessed in individual studies. ’t Hart et al. evaluated variants in *CTRB1/2* rs7202877, *CHST3* rs4148941, and *TMEM114* rs7202633 in patients with T2DM treated with a GLP-1RA for at least 3 months. None of these variants showed a significant association with a change in HbA1c [35]. Moreover, Gavriilidis et al. analyzed *CETP* rs708272 in patients treated with dulaglutide for 6 months, but this variant was not associated with HbA1c response [15]. Similarly, Pereira et al. found no significant associations between *IL6R* rs2228145 and body weight change after exenatide plus dapagliflozin treatment [31].

#### 3.2.2. Stratified Analysis by Responders vs. Non-Responders

Several studies classified patients according to predefined clinical response thresholds and subsequently compared genotype or allele distributions between responders and non-responders. However, the criteria used to define response were not uniform across studies. Response was mostly based on glycemic outcomes, including the achievement of HbA1c below 7.0%, the reduction in baseline HbA1c by at least 1.0%, or the maintenance of HbA1c below 7.0% after GLP-1RA initiation, either alone or in combination. In studies evaluating weight-related response, responders were generally defined as patients achieving a reduction of at least 3% of baseline body weight. Other studies used study-specific definitions, such as classification into optimal/suboptimal glycemic responders or strong/poor weight-loss responders. This responder-based approach was applied to variants in *GLP1R*, *CTRB1/2*, *TCF7L2*, and *PPARD*, as well as to multigenic predictive models (Table 3).

***GLP1R* variants**

Variants in *GLP1R* were evaluated in relation to both glycemic and weight-loss responder status. Eghbali et al. assessed two *GLP1R* variants, rs6923761 G>A and rs10305420 C>T, in patients treated with liraglutide for 6 months, classified as optimal or suboptimal glycemic responders. In genetic association models, homozygosity for the T allele of rs10305420 was associated with optimal glycemic response to liraglutide compared with heterozygous and wild-type homozygous states. This association was observed in both the recessive model and the codominant model. By contrast, no significant association was found between *GLP1R* rs6923761 and HbA1c reduction [19].

Jensterle et al. also evaluated *GLP1R* rs6923761 and rs10305420, but in relation to weight-loss response after liraglutide treatment, in patients classified as strong responders (n = 20, mean weight loss of 7.38 ± 1.74 kg) or poor responders (n = 37, mean weight loss of 2.11 ± 2.17 kg). Carriers of at least one rs10305420 T allele had a lower likelihood of being classified as strong responders than carriers of two C alleles [32].

***CTRB1/2* and *TCF7L2* variants**

The *CTRB1/2* rs7202877 T>G variant was investigated in relation to responder status in patients treated with GLP-1RAs. Kyriakidou et al. evaluated patients treated with liraglutide for at least 6 months in combination with oral glucose-lowering agents and/or insulin [25]. Carriers of the rs7202877 G allele showed similar responses to liraglutide compared with non-carriers, both for glycemic control and weight loss.

In a subsequent expanded study, Kyriakidou et al. assessed not only *CTRB1/2* rs7202877 T>G, but also *TCF7L2* rs7903146 C>T and *GLP1R* rs367543060 C>T in patients treated with liraglutide, exenatide, or lixisenatide, in combination with other glucose-lowering medications. Similarly, no significant differences in the distribution of the analyzed *TCF7L2*, *CTRB1/2*, or *GLP1R* variants were observed between responders and non-responders, either for glycemic control or body weight response [18].

***PPARD* variants**

Song et al. evaluated *PPARD* rs2016520 T>C and *PPARD* rs3777744 A>G in patients treated with exenatide for 6 months. Although this study was also included in the genotype-stratified analysis, the authors additionally compared genotype and allele frequencies between responders and non-responders based on the reduction in BMI and HbA1c. The *PPARD rs3777744* variant was associated with responder status. Carriers of the A allele showed a higher response rate to exenatide treatment, and AA homozygotes had the highest response rate. Specifically, the response rate was 84.00% among AA homozygotes, compared with 61.36% among AG heterozygotes and 54.55% among GG homozygotes. In contrast, no significant association of *PPARD* rs2016520 with responder status was observed [21]. These findings are consistent with the genotype-stratified results from the same study, in which rs3777744 was associated with differential changes in HbA1c and HOMA-IR [21].

**Multigenic responder models**

Beyond single-variant analyses, Villikudathil et al. applied a multi-omic and computational approach to distinguish GLP-1RA responders from non-responders related to HbA1c achievements. The study identified five genomic variants whose allelic distributions differed significantly between responders and non-responders: rs6724083, rs7596517, rs7593167, rs12911054, and rs2569431. These variants were located in or near genes including *NCKAP5*, *DPH6-AS1*, and *CTU1* [16]. In addition, 45 proteomic markers were identified as contributing to the discrimination between response groups. A machine-learning model integrating genomic and proteomic features yielded a reported classification accuracy of approximately 95% for differentiating GLP-1RA responders from non-responders in an exploratory dataset. These findings suggest that multigenic or multi-omic signatures may improve response prediction compared with analyses based on individual genetic variants, although external validation is required [16].

#### 3.2.3. Genome-Wide Association Studies

Genome-wide association studies (GWAS) have provided a broader perspective on the genetic architecture of GLP-1RA response by moving beyond predefined candidate genes and enabling the identification of novel *loci* potentially associated with therapeutic efficacy. In contrast to genotype-stratified candidate-gene studies, these analyses allow an agnostic assessment of common and low-frequency genetic variation across the genome. However, GWAS evidence remains limited, with few studies available and most associations requiring further replication and functional validation.

A GWAS meta-analysis identified genetic variations in *GLP1R* and *ARRB1* as relevant variants associated with glycemic response to GLP-1RA therapy. Dawed et al. reported that *GLP1R* rs6923761 was associated with HbA1c reduction [20]. In addition, the authors identified low-frequency variants in *ARRB1*, with the association largely driven by rs140526338, as variants associated with HbA1c response.

### 3.3. Genetic Variant Influence on Adverse Effects Related with GLP-1RA Administration

The pharmacogenetic analysis of adverse effects associated with GLP-1RA treatment was notably underrepresented across the included studies. Of the 21 studies reviewed, only three reported data on treatment-emergent adverse effects, and none did so as a primary pharmacogenetic endpoint. Ferreira et al. described that the most common adverse effects of exenatide were gastrointestinal (GI) in nature, namely nausea, vomiting, and abdominal pain; six patients discontinued treatment due to these effects, with three belonging to each TCF7L2 rs7903146 genotype group (CC and CT/TT), suggesting no genotype-related imbalance in tolerability [28]. Similarly, Guan et al. reported that GI adverse drug reactions did not differ significantly across *GLP1R* genotype groups in patients receiving exenatide or liraglutide [23]. Jensterle et al., in a study conducted in women with PCOS, reported the frequency and severity of adverse effects, predominantly nausea (15/57 patients), transient diarrhea (17/57), headache (6/57), insomnia (4/57), hypoglycemic events (4/57), and a single case of rash (1/57), and found these to be similarly distributed between strong and poor weight-loss responders, independently of *GLP1R* genotype [32]. In the remaining 18 studies, adverse effect data were either not collected or not reported in relation to the studied variants.

## 4. Discussion

This comprehensive review synthesized over a decade of worldwide literature exploring the influence of genetic variants on the therapeutic efficacy and safety of GLP-1RAs in patients with T2DM and/or obesity. Genotype-stratified studies investigated several candidate variants potentially associated with differential response to GLP-1RA therapy. The most frequently investigated associations involved *GLP1R* variants, followed by variants in genes related to β-cell function, insulin resistance, lipid and hepatic metabolism. However, the evidence was heterogeneous, with several associations reported in single studies only or showing inconsistent findings across cohorts, GLP-1RA agents, treatment durations, and clinical outcomes. Table 4 summarizes the genetic variants evaluated across the included studies, classifying each according to the overall consistency of the findings, when available from more than one study. Findings reported in a single study, as discussed below, should be considered as exploratory or hypothesis-generating.

Many studies evaluated genetic variants in the gene that codes the receptor of endogenous GLP-1, including overall five different variants in the *GLP1R* gene (rs10305420, rs6923761, rs3765467, rs1042044 and rs367543060). Globally, the evidence for *GLP1R* variants suggests possible genotype-dependent modulation of both glycemic and weight-related responses to GLP-1RAs, although findings were not fully consistent across studies. At least 1 study showed differences in rs10305420 regarding both glycemic control and BMI-related outcomes [29], and in rs3765467 regarding HbA1c reductions but not in body weight reduction [23]. Nevertheless, the greatest amount of evidence establishes no statistically significant differences between genotypes both in these and other additionally investigated genetic variants in the *GLP1R* gene [22,30,31,33]. Importantly, additional recent evidence has further highlighted the complexity of the association between *GLP1R* rs6923761 and GLP-1RA response. In individuals with severe obesity treated with semaglutide over 4 months, Phan et al. reported significantly greater weight-loss kinetics among homozygous carriers of the A allele, with sex emerging as an additional independent predictor of the response [36]. Similarly, Masha et al. observed a gene–dose association of the A allele with liraglutide-induced weight loss, despite reporting attenuated improvements in fasting insulin, glucagon, and HOMA-IR among variant carriers, although without effects on fasting glycemia [37]. Conversely, Tonin et al. found that carriers of the G allele achieved greater HbA1c reductions during treatment with GLP-1RAs (either semaglutide, dulaglutide or liraglutide), suggesting a more favorable glycemic response than carriers of the A allele [38]. Together, these findings suggest that the influence of rs6923761 may differ according to the clinical endpoint evaluated, with the A allele appearing to favor weight-loss outcomes, as found by De Luis et al. (Table 2) [33], while potentially exerting less favorable effects on glycemic and metabolic responses.

In an inverse approach, responder versus non-responder analyses investigated several variants potentially associated with differential clinical response to GLP-1RA therapy. Among *GLP1R* variants, rs10305420 showed associations with both glycemic and weight-loss responder status, although the direction and clinical interpretation differed according to the outcome assessed [19,32]. In addition, recent evidence from a large GWAS of semaglutide- and tirzepatide-treated individuals further highlights the persistent heterogeneity across studies regarding the direction of the observed effects [39]. Su et al. identified rs10305420 associated with treatment-induced weight loss, with each copy of the T allele conferring an additional 0.64% BMI reduction, and concluded that this variant was the most likely causal variant within the associated locus. These findings indeed contrast with some earlier studies included in this review, in which T-allele carriage was associated with poorer weight-loss response to liraglutide [32] or attenuated glycemic improvement with exenatide [29], whereas Eghbali et al. associated rs10305420 T-allele homozygosity with a more favorable glycemic response [19]. These findings reinforce that the relationship between *GLP1R* rs10305420 and GLP-1RA response may differ according to the clinical endpoint evaluated. Moreover, even when evaluating the same primary outcomes, these studies often included different GLP-1RAs and variable treatment durations, which complicates the interpretation and synthesis of the available evidence (Table 4).

Another GWAS supported the previous candidate-gene evidence implicating variation in the *GLP1R* in pharmacological response [20]. Nevertheless, while *GLP1R* remains the most directly implicated gene, using an unbiased approach, the authors identified *ARRB1* and additional suggestive *loci*, indicating that treatment response is likely polygenic and influenced by pathways extending beyond receptor sequence variation alone. *ARRB1* encodes β-arrestin 1, a protein involved in G protein-coupled receptor signaling and receptor desensitization [40], suggesting a potential role in modulating downstream GLP-1R signaling. The identification of *ARRB1* highlights the possibility that interindividual variability in GLP-1RA response may depend not only on variations in the receptor itself but also on genes involved in intracellular signaling pathways [20]. Variants in *ARRB1* may influence the duration and magnitude of the therapeutic response, offering a biologically plausible mechanism that warrants further investigation in the context of personalized GLP-1RA therapy.

Importantly, besides the predetermined primary outcomes (glycemic efficacy and weight-related efficacy), there were additional secondary and exploratory outcomes in the different studies. The number contributing to each outcome domain were distributed as follows: glycemic efficacy (n = 19), weight-related efficacy (n = 13), hepatic or metabolic outcomes (n = 3), mechanistic outcomes including β-cell function and insulin secretion (n = 3), and safety or tolerability (n = 3). Indeed, genetic variants in the *TCF7L2* gene were the second most studied. Transcription factor 7-like 2 regulates pancreatic beta cell mass and insulin secretion [41,42]. This interest is justified by the fact that variants in *TCF7L2*, namely the T risk allele of the rs7903146, are among the strongest genetic risk factors for T2DM, as they are associated with impaired insulin secretion [43,44,45]. Moreover, TCF7L2, which is a transcription factor downstream of the canonical Wnt signaling pathway, was shown to regulate GLP-1 production and the expression of GLP-1R in pancreatic β-cells [46]. In one of four different studies, patients carrying *TCF7L2* rs7903146 CC genotype showed decreased body weight after treatment with liraglutide [22], considered as the lowest risk/reference genotype. However, the additional studies did not find differences [18,28,30,31]. The encountered discrepancies may be partially justified by differences in treatment duration, background therapy, and outcome definition.

While *GLP1R*, *ARRB1*, and *TCF7L2* gene products are directly involved in incretin receptor signaling and downstream signal transduction, other genetic variants are more likely to influence treatment outcomes indirectly. Metabolic dysfunction-associated steatotic liver disease (MASLD), formerly denominated as NAFLD, often coexist with obesity and other associated comorbidities, such as T2DM [47,48]. Of note, GLP-1RAs such as semaglutide have demonstrated improvements in both hepatic steatosis and fibrosis in patients with MASLD [49]. Adiponutrin/PNPLA3 is involved in hepatocyte triglyceride handling. It was described that the *PNPLA3* I148M variant (carriers of the minor G allele in rs738409) reduces the rate of triglyceride hydrolysis in human hepatocytes, contributing to excessive liver fat accumulation and consequently progression to MASLD [50,51]. Evaluated in three different studies, the available evidence suggests that *PNPLA3* rs738409 may only influence selected metabolic or hepatic responses to specific GLP-1RAs. The identified associations appear to be outcome-, sex-, and treatment-dependent [15,17,27]. Besides the higher HbA1c reduction in rs738409 (C>G) genotype, hepatic steatosis scores such as fatty liver index (FLI) and hepatic steatosis index (HSI) were not different between groups in response to 6 months of dulaglutide treatment [15]. However, Urias and colleagues found that ALT levels declined more markedly in individuals carrying at least one PNPLA3 risk allele after 1 year, but not after 6 months, of semaglutide treatment [17], which reflects not only the importance of the treatment duration, but also the distinct structural and pharmacokinetic characteristics of individual GLP-1RAs. Based on these results, the potential relevance of PNPLA3 genetic variants to GLP-1RA pharmacogenetics appears to be mediated mainly through its role in hepatic lipid metabolism.

Additional genes were explored, namely *PPARD*, which encodes the protein peroxisome proliferator-activated receptor delta (PPARβ/δ). It was previously described that agonist-induced activation of PPARβ/δ stimulated *GLP-1R* expression in pancreatic β cells under lipotoxic conditions [52]. Independently of using a genotype- or responsiveness-stratified analysis, Song and colleagues found that *PPARD* rs3777744 was associated with exenatide response, with the highest response rate in terms of HbA1c reduction observed among AA homozygotes [21].

From an analysis of seven variants in five different genes (*GLP1R*, *TCF7L2*, *KCNQ1*, *WFS1* and *IL6R*), only the *WFS1* variant (rs10010131 A allele) was significantly associated with additional decrease in body weight, while no differences were found in all other genetic variants studied [31]. Despite not being considered a canonical component of the GLP-1 signaling cascade, Wolfram Syndrome 1 (WFS1) protein supports beta-cell survival by promoting insulin biosynthesis and suppressing endoplasmic reticulum (ER) stress [53]. In fact, genetic variation in WFS1 may indirectly modify the response to GLP-1RAs by influencing β-cell resilience and cell survival pathways, thereby affecting the capacity of pancreatic β-cells to benefit from GLP-1RA-mediated protective and insulinotropic effects, as described for Wolfram syndromes caused by WFS1 mutations [54]. While previous studies showed that rs10010131 was associated with reduced glucose-induced insulin secretion [55], the authors included exclusively patients with obesity without T2DM, so glycemic control was an outcome not considered in the cohort [31].

Investigating additional and diverse *loci* may allow for a more holistic approach to understanding why clinical responses to GLP-1RA extend far beyond glycemic control to encompass systemic metabolic restoration, allowing the expansion towards systemic cardiometabolic regulation, the role of inflammation, pancreatic homeostasis and adipose tissue remodeling. However, *CTRB1/2* rs7202877, *CHST3* rs4148941, *TMEM114* rs7202633, *KCNQ1* rs151290, *IL6R* rs2228145, *CNR1* rs1049353 and *CETP* rs708272 were not significantly associated with responder status in the available studies.

Interesting findings suggest that combined genetic and epigenetic profiling may provide additional predictive information beyond genotype alone. The observation of an association between a specific risk allele and promoter methylation [26] shifts the paradigm from purely structural genetic determination to dynamic transcriptional regulation. Epigenetic modifications, such as DNA methylation, may act as a bridge between environmental factors (e.g., diet and baseline metabolic state) and *GLP1R* expression or signaling. Therefore, the integration of pharmacoepigenetics [56] may represent a promising approach to improve the prediction of GLP-1RAs efficacy. However, any potential improvement in predictive accuracy compared with genetic information alone remains hypothetical and requires prospective and external validation.

Regarding the specificity of the GLP-1RA in each study, these were not always disclosed, or different molecules were evaluated together indiscriminately, which introduces a confounding factor, as molecular differences between agents could interact differently with specific receptor variants. Of note, the predominance of exenatide and liraglutide across the reviewed literature is chronologically justified by their pioneering status in the pharmaceutical market (approval in 2006 and in 2010 by EMA, respectively) [57]. Consequently, early pharmacogenetic cohorts were inherently restricted to these first-generation molecules. These possess distinct pharmacokinetic and pharmacodynamic profiles compared to contemporary weekly agents, such as semaglutide or the dual GIP/GLP-1RA tirzepatide [58]. Indeed, differences in the molecular structure, receptor binding characteristics, duration of receptor activation, dosing frequency, and clinical efficacy may influence the potential pharmacogenetic associations. Therefore, evidence derived predominantly from studies of exenatide and liraglutide should be interpreted with caution and cannot be assumed to be directly generalizable to newer GLP-1RAs or multiple agonists. Further pharmacogenetic studies involving these more recent agents are needed.

The available evidence was insufficient to establish a potential pharmacogenetic association with tolerability. Across the three studies identified showing safety-directed results, genetic variants did not appear to significantly associate with the incidence of gastrointestinal adverse effects [23,28,32]. However, given the very limited number of studies evaluating safety outcomes, the pharmacogenetics of GLP-1RAs tolerability remains largely unevaluated. The near-absence of pharmacogenetic safety data is a substantial limitation, particularly given the well-established gastrointestinal side effect profile of this drug class and the known interindividual variability in tolerability [59,60,61,62]. Recent data nevertheless suggest that pharmacogenetic influences on GLP-1RA tolerability may exist. In a large GWAS of patients treated with semaglutide or tirzepatide, Su et al. identified genome-wide significant associations between variants located near *GLP1R* and treatment-related vomiting (rs11760106) and nausea (rs9357296), two of the most common adverse effects associated with GLP-1Ras [39]. Whether additional specific genetic variants are associated with susceptibility to adverse effects of GLP-1RA, as has been suggested for other drug classes [63,64,65], remains an open and clinically relevant question that warrants dedicated investigation in future pharmacogenetic studies.

Overall, the available literature comprises a combination of isolated candidate-gene associations, predominantly null findings, and associations with inconsistent direction across studies. Several results were derived from relatively small cohorts, post hoc analyses, or studies assessing multiple variants and clinical outcomes, and most have not undergone independent replication or external validation. Consequently, statistical significance in an individual study should not be interpreted as evidence of clinical validity or utility. Even for the most frequently studied variants, including those in *GLP1R*, *TCF7L2*, and *PNPLA3*, findings varied and did not allow further comparisons according to the GLP-1RA investigated, treatment duration, concomitant therapy, population characteristics, or primary clinical endpoint (Table 1, Table 2, Table 3 and Table 4). In addition, important clinical and methodological variables, including dose-escalation protocols and adherence, or changes in concomitant medications, were inconsistently reported across studies, limiting the assessment of their potential confounding or effect-modifying influence on pharmacogenetic associations. The substantial heterogeneity in these factors may, at least in part, explain the lack of reproducibility and the inconsistent direction of the effects observed. The available evidence is therefore better interpreted as a map of potential pharmacogenetic signals and research gaps than as confirmation of reproducible response biomarkers.

A major strength of the current literature is its longitudinal and global scope, spanning over a decade of research across diverse geographical cohorts. However, several methodological caveats limit the immediate clinical translation of these findings. Most of the reviewed studies rely on retrospective observational designs with relatively small sample sizes, which inherently restricts statistical power and increases the risk of confounding variables. Furthermore, heterogeneity in treatment duration constitutes a significant hurdle: only 4 out of 21 studies evaluated outcomes beyond 6 months. Particularly for weight loss, achieving stabilization under GLP-1RA therapy requires long-term monitoring to fully observe plateau effects [66], so relying on relatively short-term data may mask genetic influences that may only manifest during sustained treatment. Importantly, although the studies analyzed included a wide diversity of geographic origins of the populations (Table 1), ethnicity and ancestry were reported inconsistently, limiting conclusions regarding population diversity and ancestry-specific pharmacogenetic effects. However, recent evidence has begun to address these gaps. In the GWAS of individuals treated with semaglutide or tirzepatide, previously mentioned, Su et al. reported ancestry-related differences in treatment efficacy, with greater weight-loss responses observed among individuals of European ancestry compared with those of Latino and African American ancestry [39]. Conversely, German et al., in a multi-ancestry analysis including 10,960 individuals from nine biobanks across six countries, observed only modest ancestry-related differences after adjustment for baseline characteristics and medication type [67]. Although individuals of African and admixed American ancestry exhibited slightly lower weight loss than those of European ancestry, the authors concluded that GLP-1RAs were effective across ancestry groups. Taken together, these recent studies reinforce both the importance of considering ancestry and population diversity in pharmacogenetic research and the continued uncertainty regarding the magnitude and clinical relevance of genetic effects on GLP-1RA response.

While the clinical promise of personalized medicine for the use of GLP-1RAs remains substantial, the translation of these findings into routine clinical practice is still far from implementation. A major confounding factor identified across the literature is the lack of a standardized definition for treatment response allowing stratified analyses. When comparing ‘responders’ versus ‘non-responders,’ the included studies relied on heterogeneous clinical endpoints. For instance, some authors defined a positive response strictly through glycemic metrics (e.g., either a threshold reduction in HbA1c % or reaching a specific target), while others focused exclusively on anthropometric outcomes, setting thresholds at BMI reduction from baseline. This phenotypic divergence across protocols severely impairs the comparability of the datasets.

Finally, although a rigorous and systematized search strategy was deployed to capture the available literature, this work does not constitute a formal systematic review. Therefore, the possibility that some relevant studies were not identified cannot be completely excluded. Moreover, no formal risk-of-bias assessment or quality appraisal of the included studies was performed, and the review does not provide certainty-of-evidence ratings or comparative methodological weighting of individual studies. The data and conclusions presented herein rely strictly on the primary analysis provided by the original authors. Given the high heterogeneity in potential confounding factors and varying definitions of clinical outcomes across the original articles, the reported associations should be interpreted as a characterization of the available evidence rather than as confirmation of clinical validity or utility. This underscores the need for caution when generalizing specific effect sizes and highlights the necessity for future, high-powered meta-analyses to formally pool these pharmacogenetic data. To establish whether a genetic variant specifically modifies the effect of GLP-1RA therapy, appropriately controlled studies incorporating untreated or active-comparator groups and formal genotype-by-treatment interaction analyses will be required. From a clinical perspective, no genetic variant identified in the current literature has sufficiently consistent, independently replicated, and externally validated evidence to support routine pharmacogenetic testing or genotype-guided prescribing of GLP-1RAs. The associations mapped in this review should therefore be regarded as research findings rather than clinically actionable biomarkers.

## 5. Conclusions

The candidate genes investigated across the literature can be broadly categorized into two biological groups: genes directly related to the pharmacological target of GLP-RAs and genes involved in the pathophysiology of metabolic disease. Unsurprisingly, the *GLP1R* gene remains the most scrutinized target, as it encodes the receptor targeted by GLP-1RAs, and genetic variation may affect receptor properties or downstream signaling. Other genes present roles in beta-cell function and insulin secretion, lipid metabolism and hepatic steatosis, energy balance and metabolic inflammation. Despite some discrepancies in the results, the collective evidence supports a multifactorial interpretation of interindividual variability in glycemic control, weight loss, and other clinical outcomes observed during GLP-1RA treatment, involving both genetic and non-genetic factors. Among the *loci* investigated, *GLP1R* currently has the strongest cumulative evidence for genotype–outcome associations during GLP-1RA treatment, supported by multiple candidate-gene studies and genome-wide analyses, although findings for individual variants remain inconsistent across populations, GLP-1RAs, and clinical outcomes. Importantly, no individual genetic variant has yet demonstrated sufficiently consistent, replicated, and clinically validated associations to support routine genotype-guided selection of GLP-1RA therapy, and the available evidence generally does not establish treatment-specific genetic effect modification. Larger, adequately powered prospective studies with standardized outcome definitions and validation across diverse populations and individual GLP-1RAs are therefore required before pharmacogenetic information can be incorporated into clinical decision-making.

## Figures and Tables

**Table 1:** Characterization of the studies included in the review.

Author, Year [Ref]	Country	Sample Size	GLP-1RA	Treatment Duration
Gavriilidis S, 2024 [15]	Greece	80	Dulaglutide	6 months
Villikudathil AT, 2024 [16]	Northern Ireland, UK	85	Not specified	NR
Urias E, 2024 [17]	USA	220	Semaglutide	1.6 years
Kyriakidou A, 2024 [18]	Greece	191	Liraglutide, Exenatide, Lixisenatide	≥6 months
Eghbali M, 2024 [19]	Iran	233	Liraglutide	6 months
Dawed AY, 2023 [20]	Multi-national	4571	Exenatide, Liraglutide, Dulaglutide, Albiglutide, Lixisenatide, Semaglutide	6 months
Song J, 2022 [21]	China	300 (genotyping); 105 (pharmacogenetic analysis)	Exenatide	6 months
Maselli D, 2022 [22]	USA	136 (Liraglutide: 67; Placebo: 69)	Liraglutide	4 months
Guan Z, 2022 [23]	China	176 (Exenatide: 64; Liraglutide: 112)	Exenatide, Liraglutide	3 months
Geng Z, 2022 [24]	China	100	Exenatide	12 months
Kyriakidou A, 2022 [25]	Greece	116	Liraglutide	≥6 months
Lin C-H, 2021 [26]	Taiwan	138 (Training: 10; Validation: 128)	Liraglutide, Exenatide, Dulaglutide	3 months
Chen Y, 2020 [27]	China	10	Exenatide	6 months
Ferreira MC, 2019 [28]	Brazil	56 (completed: 46)	Exenatide	2 months
Yu M, 2019 [29]	China	285	Exenatide	6 months
Chedid V, 2018 [30]	USA	59 (Liraglutide: 18; Exenatide: 10; Placebo: 31)	Liraglutide, Exenatide	1 month (Exenatide); ~1.25 months (Liraglutide)
Pereira MJ, 2018 [31]	Sweden	50	Exenatide	6 months
Jensterle M, 2015 [32]	Slovenia	32 (Liraglutide: 17; Metformin: 15)	Liraglutide	3 months
de Luis DA, 2015 [33]	Spain	90	Liraglutide	~3.2 months
de Luis DA, 2014 [34]	Spain	86	Liraglutide	~3.2 months
‘t Hart LM, 2013 [35]	Netherlands	173	Not specified	≥3 months

Abbreviations: GLP-1RA, glucagon-like peptide-1 receptor agonist; NR, not reported; RCT, randomized controlled trial.

**Table 2:** GLP-1RA response stratified by genotype.

Author, Year [Ref]	GLP-1RA	Gene	Genetic Variant(s)	Glycemic Control Outcomes	BMI-Related Outcomes
Gavriilidis S, 2024 [15]	Dulaglutide	*PNPLA3*	rs738409 (C>G, I148M)	CC and CG genotypes: greater HbA1c reduction vs. GG homozygotes at 6 months (CC −0.93%, CG −0.64%, GG +0.39%); effect mainly observed in females	NR
		*CETP*	rs708272 (TaqIB)	No significant association with HbA1c response	NR
Urias E, 2024 [17]	Semaglutide	*PNPLA3*	rs738409 (C>G)	NR	BMI change similar across genotype groups (CC: −7.0% vs. CG/GG: −6.3%; *p* = 0.93); no significant genotype × BMI interaction
Song J, 2022 [21]	Exenatide	*PPARD*	rs2016520 (T>C)	No significant association with HbA1c reduction; C-allele carriers: greater HOMA-IR reduction vs. TT	No significant association with BMI change
			rs3777744 (A>G)	AA genotype: greater HbA1c reduction and HOMA-IR improvement vs. AG/GG; AG/GG: attenuated HbA1c and FPG response	No significant association with BMI change
Maselli D, 2022 [22]	Liraglutide	*GLP1R*	rs6923761 (G>A)	NR	Trend toward lower total body fat % in AG/AA vs. GG (*p* = 0.062); no significant difference in body weight
		*TCF7L2*	rs7903146 (C>T)	NR	CC genotype vs. CT/TT genotypes: lower body weight at 16 weeks (*p* = 0.015), similar at baseline (*p* = 0.435)
Guan Z, 2022 [23]	Exenatide, Liraglutide	*GLP1R*	rs10305420 (C>T)	No significant differences in HbA1c reduction	No significant between-group differences in body weight
			rs3765467 (G>A)	GG genotype: greater HbA1c reduction vs. GA/AA (−1.7 ± 2.4% vs. −0.8 ± 1.8%, *p* = 0.002)	No significant between-group differences in body weight
Geng Z, 2022 [24]	Exenatide	*KCNQ1*	rs163184 (T>G)	TG/GG genotypes: smaller HbA1c reduction (−0.34%, 95% CI −0.61 to −0.06) and FPG reduction (−0.6, 95% CI −1.07 to −0.13) vs. TT	No significant association with weight loss response
Lin C-H, 2021 [26]	Liraglutide, Exenatide, Dulaglutide	*VTRNA2-1*	rs2346018 (A>C)	Allele + promoter hypomethylation (<40%) was associated with poor glycemic response (OR 4.048, 95% CI 1.438–11.389, *p* = 0.007); rs2346018 alone not independently significant	NR
Chen Y, 2020 [27]	Exenatide	*PNPLA3*	rs738409 (I148M)	HbA1c change: I148M+ −2.83 ± 0.53%; I148I −2.93 ± 0.57%; no significant between-group difference (*p* = 0.83)	BMI change: I148M+ −2.16 ± 0.67 kg/m^2^; I148I −1.17 ± 0.52 kg/m^2^; no significant between-group difference (*p* = 0.29)
Yu M, 2019 [29]	Exenatide	*GLP1R*	rs10305420 (C>T)	rs10305420: T-allele carriers: smaller HbA1c reduction (−0.4% difference, *p* = 0.002) vs. C/C homozygotes after 6 months	T-allele carriers: less weight loss (−1.27 kg difference, *p* = 0.02) vs. C/C homozygotes after 6 months
			rs3765467	No significant difference (*p* = 0.07)	No significant difference (*p* = 0.58)
Ferreira MC, 2019 [28]	Exenatide	*TCF7L2*	rs7903146 (C>T)	FPG reduced in both groups; no significant between-group difference in glucose levels during meal test	Body weight reduced in both CC (−2.16 ± 2.2 kg) and CT/TT (−2.49 ± 6.8 kg) groups; no significant between-group difference
Chedid V, 2018 [30]	Liraglutide, Exenatide	*GLP1R*	rs6923761 (G>A)	NR	No significant trend toward greater weight loss in A/G-allele carriers vs. GG (liraglutide: −6.7 ± 1.4 kg vs. −4.6 ± 1.2 kg; exenatide: −0.9 ± 0.5 kg vs. −1.9 ± 0.4 kg)
		*TCF7L2*	rs7903146 (C>T)	NR	No significant difference in weight loss between genotype groups (liraglutide: CT/TT −7.1 ± 1.9 kg vs. CC −4.7 ± 1.0 kg; exenatide: CT/TT −1.7 ± 0.4 kg vs. CC −0.5 ± 0.4 kg)
Pereira MJ, 2018 [31]	Exenatide	*GLP1R*	rs10305420 (C>T)	NR	No significant association with body weight change (*p* > 0.05)
			rs6923761	NR	No significant association with body weight change (*p* > 0.05)
			rs1042044	NR	No significant association with body weight change (*p* > 0.05)
		*TCF7L2*	rs7903146 (C>T)	NR	No significant association with body weight change (*p* > 0.05)
		*KCNQ1*	rs151290	NR	No significant association with body weight change (*p* > 0.05)
		*WFS1*	rs10010131 (G>A)	NR	A/G or A/A genotypes: greater weight loss vs. GG homozygotes (*p* < 0.05)
		*IL6R*	rs2228145	NR	No significant association with body weight change (*p* > 0.05)
de Luis DA, 2015 [33]	Liraglutide	*GLP1R*	rs6923761 (G>A)	HbA1c reduced in both groups (GG −0.8%; GA/AA −1.0%); no significant between-group difference	Greater weight, BMI, and fat mass reduction in A-allele carriers vs. GG (weight: −4.52 ± 4.6 kg vs. −2.78 ± 2.8 kg; *p* < 0.05)
de Luis DA, 2014 [34]	Liraglutide	*CNR1*	rs1049353 (G1359A)	HbA1c decreased in both genotype groups: GG −1.2% (8.6→7.4%); A allele carriers −1.0% (8.6→7.6%); no significant between-group difference	BMI, body weight, and fat mass decreased in both groups, with no significant between-group difference
‘t Hart LM, 2013 [35]	GLP-1 RA (NR)	*CTRB1/2*	rs7202877	No significant association with HbA1c response to GLP-1 RA	NR
		*CHST3*	rs4148941	No significant association with HbA1c response to GLP-1 RA	NR
		*TMEM114*	rs7202633	No significant association with HbA1c response to GLP-1 RA	NR

Abbreviations: BMI, body mass index; FPG, fasting plasma glucose; GLP-1 RA, glucagon-like peptide-1 receptor agonist; HbA1c, glycated hemoglobin; HOMA-IR, homeostatic model assessment of insulin resistance; NR, not reported; OGTT, oral glucose tolerance test; OR, odds ratio; WHR, waist-to-hip ratio.

**Table 3:** GLP-1RA response stratified by responders vs. non-responders.

Author, Year [Ref]	GLP-1RA	Clinical Response Thresholds (Responders’ Definition)	Gene	Genetic Variant(s)	Glycemic Control Outcomes	BMI-Related Outcomes
Villikudathil AT, 2024 [16]	NR	HbA1c < 53 mmol/mol (≈7%): responders; HbA1c ≥ 53 mmol/mol: non-responders	*NCKAP5*	rs6724083, rs7596517, rs7593167	Allele distributions of rs6724083, rs7596517, rs7593167 significantly differed between responders and non-responders; included in exploratory machine-learning model; reported classification accuracy of around 95%, without independent external validation	NR
			*DPH6-AS1*	rs12911054	Allele distribution significantly differed between responders and non-responders; included in machine-learning model	NR
			*CTU1*	rs2569431	Allele distribution significantly differed between responders and non-responders; included in machine-learning model	NR
Kyriakidou A, 2024 [18]	Liraglutide, Exenatide, Lixisenatide	Glycemic response: HbA1c < 7%, or reduction ≥ 1% from baseline, or maintenance of HbA1c < 7% at 3 or 6 months. Weight-loss response: body weight reduction ≥ 3% of baseline at 3 or 6 months (NICE criteria)	*TCF7L2*	rs7903146 (C>T)	No significant association with glycemic responder status (OR 1.08, 95% CI 0.50–2.31, *p* = 0.85)	No significant association with weight-loss responder status (OR 1.40, 95% CI 0.56–3.47, *p* = 0.47)
			*CTRB1/2*	rs7202877 (T>G)	No significant association with glycemic responder status (OR 1.35, 95% CI 0.66–2.76, *p* = 0.42)	No significant association with weight-loss responder status (OR 1.28, 95% CI 0.55–2.98, *p* = 0.57)
			*GLP1R*	rs367543060 (C>T)	No significant association with glycemic responder status	No significant association with weight-loss responder status
Eghbali M, 2024 [19]	Liraglutide	Optimal glycemic responders: HbA1c reduction > median reduction within each baseline HbA1c category (7–7.99%, 8–8.99%, 9–9.99%, ≥10%). Suboptimal responders: HbA1c reduction ≤ median	*GLP1R*	rs10305420 (C>T)	TT homozygosity associated with optimal glycemic response (recessive model adjusted OR 3.42, 95% CI 1.44–8.10, *p* = 0.005); median HbA1c reduction: optimal −2.5% vs. suboptimal −1.0%	NR
				rs6923761 (G>A)	No significant association with glycemic responder status	NR
Song J, 2022 [21]	Exenatide	Responders: HbA1c reduction ≥ 1% or body weight reduction ≥ 3% after 6 months (NICE-based criteria)	*PPARD*	rs2016520 (T>C)	No significant association with responder status	No significant association with responder status
				rs3777744 (A>G)	AA homozygotes: highest response rate (84.0%); AG: 61.4%; GG: 54.5% (*p* = 0.022); A-allele carriers: higher overall response rate (*p* = 0.007)	No significant association with weight-loss responder status
Kyriakidou A, 2022 [25]	Liraglutide	Glycemic response: HbA1c < 7%, or reduction ≥1% from baseline, or maintenance of HbA1c < 7% after switching to liraglutide. Weight-loss response: body weight reduction ≥ 3% of baseline (NICE criteria)	*CTRB1/2*	rs7202877 (T>G)	No significant association with glycemic responder status	No significant association with weight-loss responder status
Jensterle M, 2015 [32]	Liraglutide	Strong responders: body weight loss ≥5% of initial body weight. Poor responders: weight loss <5%	*GLP1R*	rs6923761 (G>A)	NR	No significant association with weight-loss responder status
				rs10305420 (C>T)	NR	Carriers of ≥1 T allele: lower likelihood of strong weight-loss response vs. wild-type homozygotes (OR 0.27, 95% CI 0.09–0.85, *p* = 0.025)

Abbreviations: BMI, body mass index; GLP-1RA, glucagon-like peptide-1 receptor agonist; HbA1c, glycated hemoglobin; NICE, National Institute for Health and Care Excellence; NR, not reported; OR, odds ratio; PPARD, peroxisome proliferator-activated receptor delta.

**Table 4:** Summary of the evidence supporting potential genetic predictors of GLP-1RA response.

Genetic Variant	Number of Studies	Sample Size (Positive/Total)	GLP-1RAs (Treatment Duration)	Overall Evidence
*GLP1R* rs10305420	5	535/761	Exenatide, Liraglutide (3–6 months)	Inconsistent
*GLP1R* rs6923761	4	90/235	Exenatide, Liraglutide (1–6 months)	Inconsistent/predominantly negative
*GLP1R* rs3765467	2	112/397	Exenatide, Liraglutide (3–6 months)	Inconsistent
*TCF7L2* rs7903146	5	67/382	Exenatide, Liraglutide, Lixisenatide (1–>6 months)	Inconsistent/predominantly negative
*PNPLA3* rs738409	3	80/230	Exenatide, Semaglutide, Dulaglutide (5–24 months)	Inconsistent/predominantly negative
*CTRB1/2* rs7202877	3	0/364 ^#^	Exenatide, Liraglutide, Lixisenatide (3–>6 months)	Negative

Variants were classified according to the consistency of findings across studies rather than the magnitude of the reported effects. Inconsistent evidence was assigned when multiple studies reported conflicting findings. Predominantly negative evidence was assigned when null findings were reported in a greater number of studies than those reporting significant genotype-associated differences. In the “Sample size” column, the numerator indicates the cumulative sample size from studies with positive findings and the denominator indicates the cumulative sample size from all studies evaluating the variant. ^#^ Although three studies investigated this genetic variant, one was an extension of a previously reported study; to avoid overlap between cohorts, only the sample size of the expanded study was included in the total sample count.

## Data Availability

This study is a review article and does not involve the generation of new data. All data discussed are cited within the manuscript and available in the referenced sources.

## Supplementary Materials

The following supporting information can be downloaded at: https://www.mdpi.com/article/10.3390/life16091486/s1, Table S1: Search strategy; Table S2: Baseline characterization of the population in each study.

## References

[1] (2023). GBD 2021 Diabetes Collaborators Global, Regional, and National Burden of Diabetes from 1990 to 2021, with Projections of Prevalence to 2050: A Systematic Analysis for the Global Burden of Disease Study 2021. Lancet.

[2] Galicia-Garcia U., Benito-Vicente A., Jebari S., Larrea-Sebal A., Siddiqi H., Uribe K.B., Ostolaza H., Martín C. (2020). Pathophysiology of Type 2 Diabetes Mellitus. Int. J. Mol. Sci..

[3] Bajaj M., McCoy R.G., Balapattabi K., Bannuru R.R., Bellini N.J., Bennett A.K., Beverly E.A., Briggs Early K., ChallaSivaKanaka S., American Diabetes Association Professional Practice Committee for Diabetes (2026). 2. Diagnosis and Classification of Diabetes: Standards of Care in Diabetes—2026. Diabetes Care.

[4] Bajaj M., McCoy R.G., Balapattabi K., Bannuru R.R., Bellini N.J., Bennett A.K., Beverly E.A., Briggs Early K., ChallaSivaKanaka S., American Diabetes Association Professional Practice Committee for Diabetes (2026). 9. Pharmacologic Approaches to Glycemic Treatment: Standards of Care in Diabetes—2026. Diabetes Care.

[5] Jastreboff A.M., Aronne L.J., Ahmad N.N., Wharton S., Connery L., Alves B., Kiyosue A., Zhang S., Liu B., Bunck M.C. (2022). Tirzepatide Once Weekly for the Treatment of Obesity. N. Engl. J. Med..

[6] Evans W.E., Johnson J.A. (2001). Pharmacogenomics: The Inherited Basis for Interindividual Differences in Drug Response. Annu. Rev. Genom. Hum. Genet..

[7] Collins R.L., Brand H., Karczewski K.J., Zhao X., Alföldi J., Francioli L.C., Khera A.V., Lowther C., Gauthier L.D., Wang H. (2020). A Structural Variation Reference for Medical and Population Genetics. Nature.

[8] Auton A., Brooks L.D., Durbin R.M., Garrison E.P., Kang H.M., Korbel J.O., Marchini J.L., McCarthy S., McVean G.A., 1000 Genomes Project Consortium (2015). A Global Reference for Human Genetic Variation. Nature.

[9] Chung W.K., Erion K., Florez J.C., Hattersley A.T., Hivert M.-F., Lee C.G., McCarthy M.I., Nolan J.J., Norris J.M., Pearson E.R. (2020). Precision Medicine in Diabetes: A Consensus Report From the American Diabetes Association (ADA) and the European Association for the Study of Diabetes (EASD). Diabetes Care.

[10] Nasykhova Y.A., Tonyan Z.N., Mikhailova A.A., Danilova M.M., Glotov A.S. (2020). Pharmacogenetics of Type 2 Diabetes-Progress and Prospects. Int. J. Mol. Sci..

[11] Fors A., Forslund T., Sundström A., Wettermark B. (2025). Prescribing Patterns of Glucagon-like Peptide-1 Receptor Agonists in the Swedish Capital Region—A Register-Based Cross-Sectional Study. Eur. J. Clin. Pharmacol..

[12] Moiz A., Filion K.B., Tsoukas M.A., Yu O.H.Y., Peters T.M., Eisenberg M.J. (2025). The Expanding Role of GLP-1 Receptor Agonists: A Narrative Review of Current Evidence and Future Directions. eClinicalMedicine.

[13] Ukhanova M., Wozny J.S., Truong C.N., Ghosh L., Krause T.M. (2025). Trends in Glucagon-like Peptide 1 Receptor Agonist Prescribing Patterns. Am. J. Manag. Care.

[14] Ouzzani M., Hammady H., Fedorowicz Z., Elmagarmid A. (2016). Rayyan—A Web and Mobile App for Systematic Reviews. Syst. Rev..

[15] Gavriilidis S., Andrianopoulou R., Ntenti C., Sarakapina A., Trakatelli C., Polyzos S.A., Goulas A. (2024). The Effect of Dulaglutide on Glycated Hemoglobin Is Associated with PNPLA3 Ι148Μ Gene Polymorphism in Patients with Type 2 Diabetes Mellitus. Endocrine.

[16] Villikudathil A.T., Mc Guigan D.H., English A. (2024). Computational Approaches for Clinical, Genomic and Proteomic Markers of Response to Glucagon-like Peptide-1 Therapy in Type-2 Diabetes Mellitus: An Exploratory Analysis with Machine Learning Algorithms. Diabetes Metab. Syndr. Clin. Res. Rev..

[17] Urias E., Tedesco N.R., Oliveri A., Raut C., Zawistowski M., Zöllner S., Speliotes E.K., Chen V.L. (2024). PNPLA3 Risk Allele Association With ALT Response to Semaglutide Treatment. Gastroenterology.

[18] Kyriakidou A., Kyriazou A.V., Koufakis T., Vasilopoulos Y., Avramidis I., Baltagiannis S., Goulis D.G., Kotsa K. (2024). Association between Variants in *TCF7L2*, *CTRB1/2*, and *GLP-1R* Genes and Response to Therapy with Glucagon-like Peptide-1 Receptor Agonists. Postgrad. Med..

[19] Eghbali M., Alaei-Shahmiri F., Hashemi-Madani N., Emami Z., Mostafavi L., Malek M., Khamseh M.E. (2024). Glucagon-Like Peptide 1 (GLP-1) Receptor Variants and Glycemic Response to Liraglutide: A Pharmacogenetics Study in Iranian People with Type 2 Diabetes Mellitus. Adv. Ther..

[20] Dawed A.Y., Mari A., Brown A., McDonald T.J., Li L., Wang S., Hong M.-G., Sharma S., Robertson N.R., Mahajan A. (2023). Pharmacogenomics of GLP-1 Receptor Agonists: A Genome-Wide Analysis of Observational Data and Large Randomised Controlled Trials. Lancet Diabetes Endocrinol..

[21] Song J., Li N., Hu R., Yu Y., Xu K., Ling H., Lu Q., Yang T., Wang T., Yin X. (2022). Effects of PPARD Gene Variants on the Therapeutic Responses to Exenatide in Chinese Patients with Type 2 Diabetes Mellitus. Front. Endocrinol..

[22] Maselli D., Atieh J., Clark M.M., Eckert D., Taylor A., Carlson P., Burton D.D., Busciglio I., Harmsen W.S., Vella A. (2022). Effects of Liraglutide on Gastrointestinal Functions and Weight in Obesity: A Randomized Clinical and Pharmacogenomic Trial. Obesity.

[23] Guan Z., Du Y., Li R., Zhang S., Xu Y., Zhang X., Zhang F., Yin Y., Wu K., Li X. (2022). Association between Glucagon-like Peptide-1 Receptor Gene Polymorphism and Treatment Response to GLP1R Agonists in Chinese Patients with Type 2 Diabetes: A Prospective Cohort Study. Eur. J. Clin. Pharmacol..

[24] Geng Z., Li Q., Huang R., Wang J., Weng J., Zhou K., Liang H., Wang Y. (2022). *KCNQ1* Variant Rs163184 Is a Potential Biomarker of Glycemic Response to Exenatide. Pharmacogenomics.

[25] Kyriakidou A., Kyriazou A.V., Koufakis T., Vasilopoulos Y., Grammatiki M., Tsekmekidou X., Avramidis I., Baltagiannis S., Goulis D.G., Zebekakis P. (2022). Clinical and Genetic Predictors of Glycemic Control and Weight Loss Response to Liraglutide in Patients with Type 2 Diabetes. J. Pers. Med..

[26] Lin C.-H., Lee Y.-S., Huang Y.-Y., Tsai C.-N. (2021). Methylation Status of Vault RNA 2-1 Promoter Is a Predictor of Glycemic Response to Glucagon-like Peptide-1 Analog Therapy in Type 2 Diabetes Mellitus. BMJ Open Diabetes Res. Care.

[27] Chen Y., Yan X., Xu X., Yuan S., Xu F., Liang H. (2020). PNPLA3 I148M Is Involved in the Variability in Anti-NAFLD Response to Exenatide. Endocrine.

[28] Ferreira M.C., da Silva M.E.R., Fukui R.T., do Carmo Arruda-Marques M., Azhar S., Dos Santos R.F. (2019). Effect of TCF7L2 Polymorphism on Pancreatic Hormones after Exenatide in Type 2 Diabetes. Diabetol. Metab. Syndr..

[29] Yu M., Wang K., Liu H., Cao R. (2019). GLP1R Variant Is Associated with Response to Exenatide in Overweight Chinese Type 2 Diabetes Patients. Pharmacogenomics.

[30] Chedid V., Vijayvargiya P., Carlson P., Van Malderen K., Acosta A., Zinsmeister A., Camilleri M. (2018). Allelic Variant in the Glucagon-like Peptide 1 Receptor Gene Associated with Greater Effect of Liraglutide and Exenatide on Gastric Emptying: A Pilot Pharmacogenetics Study. Neurogastroenterol. Motil..

[31] Pereira M.J., Lundkvist P., Kamble P.G., Lau J., Martins J.G., Sjöström C.D., Schnecke V., Walentinsson A., Johnsson E., Eriksson J.W. (2018). A Randomized Controlled Trial of Dapagliflozin Plus Once-Weekly Exenatide Versus Placebo in Individuals with Obesity and Without Diabetes: Metabolic Effects and Markers Associated with Bodyweight Loss. Diabetes Ther..

[32] Jensterle M., Kravos N.A., Pfeifer M., Kocjan T., Janez A. (2015). A 12-Week Treatment with the Long-Acting Glucagon-like Peptide 1 Receptor Agonist Liraglutide Leads to Significant Weight Loss in a Subset of Obese Women with Newly Diagnosed Polycystic Ovary Syndrome. Hormones.

[33] de Luis D.A., Diaz Soto G., Izaola O., Romero E. (2015). Evaluation of Weight Loss and Metabolic Changes in Diabetic Patients Treated with Liraglutide, Effect of RS 6923761 Gene Variant of Glucagon-like Peptide 1 Receptor. J. Diabetes Complicat..

[34] de Luis D.A., Ovalle H.F., Soto G.D., Izaola O., De La Fuente B., Romero E. (2014). Role of Genetic Variation in the Cannabinoid Receptor Gene (CNR1) (G1359A Polymorphism) on Weight Loss and Cardiovascular Risk Factors after Liraglutide Treatment in Obese Patients with Diabetes Mellitus Type 2. J. Investig. Med..

[35] ’t Hart L.M., Fritsche A., Nijpels G., van Leeuwen N., Donnelly L.A., Dekker J.M., Alssema M., Fadista J., Carlotti F., Gjesing A.P. (2013). The CTRB1/2 Locus Affects Diabetes Susceptibility and Treatment via the Incretin Pathway. Diabetes.

[36] Phan A., Carette C., Narjoz C., Rives-Lange C., Rassy N., Czernichow S., Pallet N. (2025). A *GLP1R* Gene Variant and Sex Influence the Response to Semaglutide Treatment in Patients with Severe Obesity. Obesity.

[37] Mashayekhi M., Safa B.I., Nian H., Devin J.K., Gamboa J.L., Yu C., Chen R., Beckman J.A., Koethe J.R., Silver H.J. (2025). RISING STARS: Effects of a GLP-1 Receptor Polymorphism on Responses to Liraglutide. J. Endocrinol..

[38] Tonin G., Goričar K., Blagus T., Janež A., Dolžan V., Klen J. (2025). Genetic Variability in Sodium-Glucose Cotransporter 2 and Glucagon-like Peptide 1 Receptor Effect on Glycemic and Pressure Control in Type 2 Diabetes Patients Treated with SGLT2 Inhibitors and GLP-1RA in the Everyday Clinical Practice. Front. Endocrinol..

[39] Su Q.J., Ashenhurst J.R., Xu W., Tran V., Ryanne Wu R., Weldon C.H., Shi J., Hicks B., Bell R.K., 23andMe Research Team (2026). Genetic Predictors of GLP1 Receptor Agonist Weight Loss and Side Effects. Nature.

[40] Jean-Charles P.-Y., Kaur S., Shenoy S.K. (2017). G Protein–Coupled Receptor Signaling Through β-Arrestin–Dependent Mechanisms. J. Cardiovasc. Pharmacol..

[41] Loder M.K., Xavier G.d.S., McDonald A., Rutter G.A. (2008). TCF7L2 Controls Insulin Gene Expression and Insulin Secretion in Mature Pancreatic β-Cells. Biochem. Soc. Trans..

[42] Takamoto I., Kubota N., Nakaya K., Kumagai K., Hashimoto S., Kubota T., Inoue M., Kajiwara E., Katsuyama H., Obata A. (2014). TCF7L2 in Mouse Pancreatic Beta Cells Plays a Crucial Role in Glucose Homeostasis by Regulating Beta Cell Mass. Diabetologia.

[43] Grant S.F.A., Thorleifsson G., Reynisdottir I., Benediktsson R., Manolescu A., Sainz J., Helgason A., Stefansson H., Emilsson V., Helgadottir A. (2006). Variant of Transcription Factor 7-like 2 (TCF7L2) Gene Confers Risk of Type 2 Diabetes. Nat. Genet..

[44] Lyssenko V., Lupi R., Marchetti P., Del Guerra S., Orho-Melander M., Almgren P., Sjögren M., Ling C., Eriksson K.-F., Lethagen υ.-L. (2007). Mechanisms by Which Common Variants in the TCF7L2 Gene Increase Risk of Type 2 Diabetes. J. Clin. Investig..

[45] Cropano C., Santoro N., Groop L., Dalla Man C., Cobelli C., Galderisi A., Kursawe R., Pierpont B., Goffredo M., Caprio S. (2017). The Rs7903146 Variant in the *TCF7L2* Gene Increases the Risk of Prediabetes/Type 2 Diabetes in Obese Adolescents by Impairing β-Cell Function and Hepatic Insulin Sensitivity. Diabetes Care.

[46] Chiang Y.A., Ip W., Jin T. (2012). The Role of the Wnt Signaling Pathway in Incretin Hormone Production and Function. Front. Physio..

[47] Caturano A., Erul E., Nilo R., Nilo D., Russo V., Rinaldi L., Acierno C., Akkus E., Koudelkova K., Cerini F. (2026). Metabolic Dysfunction-associated Steatotic Liver Disease, Insulin Resistance and Hepatocellular Carcinoma: A Deadly Triad. Eur. J. Clin. Investig..

[48] Jeeyavudeen M.S., Khan S.K., Fouda S., Pappachan J.M. (2023). Management of Metabolic-Associated Fatty Liver Disease: The Diabetology Perspective. World J. Gastroenterol..

[49] Malandris K., Charalampidis K., Loomba R., Sinakos E. (2026). Pharmacologic Treatment of Metabolic Dysfunction-Associated Steatotic Liver Disease in the Context of Type 2 Diabetes. Curr. Diab Rep..

[50] Perttilä J., Huaman-Samanez C., Caron S., Tanhuanpää K., Staels B., Yki-Järvinen H., Olkkonen V.M. (2012). PNPLA3 Is Regulated by Glucose in Human Hepatocytes, and Its I148M Mutant Slows down Triglyceride Hydrolysis. Am. J. Physiol.-Endocrinol. Metab..

[51] Sookoian S., Castaño G.O., Burgueño A.L., Gianotti T.F., Rosselli M.S., Pirola C.J. (2009). A Nonsynonymous Gene Variant in the Adiponutrin Gene Is Associated with Nonalcoholic Fatty Liver Disease Severity. J. Lipid Res..

[52] Yang Y., Tong Y., Gong M., Lu Y., Wang C., Zhou M., Yang Q., Mao T., Tong N. (2014). Activation of PPARβ/δ Protects Pancreatic β Cells from Palmitate-Induced Apoptosis by Upregulating the Expression of GLP-1 Receptor. Cell. Signal..

[53] Abreu D., Asada R., Revilla J.M.P., Lavagnino Z., Kries K., Piston D.W., Urano F. (2020). Wolfram Syndrome 1 Gene Regulates Pathways Maintaining Beta-Cell Health and Survival. Lab. Investig..

[54] Panfili E., Frontino G., Pallotta M.T. (2023). GLP-1 Receptor Agonists as Promising Disease-Modifying Agents in WFS1 Spectrum Disorder. Front. Clin. Diabetes Healthc..

[55] Schäfer S.A., Müssig K., Staiger H., Machicao F., Stefan N., Gallwitz B., Häring H.U., Fritsche A. (2009). A Common Genetic Variant in WFS1 Determines Impaired Glucagon-like Peptide-1-Induced Insulin Secretion. Diabetologia.

[56] Smith D.A., Sadler M.C., Altman R.B. (2023). Promises and Challenges in Pharmacoepigenetics. Camb. Prism. Precis. Med..

[57] Blind E., Janssen H., Dunder K., De Graeff P.A. (2018). The European Medicines Agency’s Approval of New Medicines for Type 2 Diabetes. Diabetes Obes. Metab..

[58] Petersen J., Finan B., Johansen V.B.I., Müller T.D., Clemmensen C. (2026). The evolving landscape of obesity pharmacotherapy. Nat. Rev. Drug Discov..

[59] Chiang C.-H., Jaroenlapnopparat A., Colak S.C., Yu C.-C., Xanthavanij N., Wang T.-H., See X.Y., Lo S.-W., Ko A., Chang Y.-C. (2025). Glucagon-Like Peptide-1 Receptor Agonists and Gastrointestinal Adverse Events: A Systematic Review and Meta-Analysis. Gastroenterology.

[60] Frazier R.D., Hasler W.L. (2025). Impact of GLP-1 Receptor Agonists on Gastrointestinal Function and Symptoms. Expert. Rev. Gastroenterol. Hepatol..

[61] Liu L., Chen J., Wang L., Chen C., Chen L. (2022). Association between Different GLP-1 Receptor Agonists and Gastrointestinal Adverse Reactions: A Real-World Disproportionality Study Based on FDA Adverse Event Reporting System Database. Front. Endocrinol..

[62] Trujillo J. (2020). Safety and Tolerability of Once-weekly GLP-1 Receptor Agonists in Type 2 Diabetes. J. Clin. Pharm. Ther..

[63] Micaglio E., Locati E.T., Monasky M.M., Romani F., Heilbron F., Pappone C. (2021). Role of Pharmacogenetics in Adverse Drug Reactions: An Update towards Personalized Medicine. Front. Pharmacol..

[64] Curless B., Momary K. (2015). Small Genetic Variants That Have a Huge Impact on Adverse Drug Reactions. Pharm. Pract. Res..

[65] Roden D.M., Wilke R.A., Kroemer H.K., Stein C.M. (2011). Pharmacogenomics: The Genetics of Variable Drug Responses. Circulation.

[66] Li Z., Han Z., Sun R., Xuan X., Huang C. (2025). Long-Term Efficacy Trajectories of GLP-1 Receptor Agonists: A Systematic Review and Network Meta-Analysis. DMSO.

[67] German J., Cordioli M., Tozzo V., Urbut S., Arumäe K., Smit R.A.J., Lee J., Li J.H., Janucik A., Ding Y. (2025). Association between Plausible Genetic Factors and Weight Loss from GLP1-RA and Bariatric Surgery. Nat. Med..

