# Supplementary material for: Genetic Variants Associated with Response to GLP-1 Receptor Agonists in Diabetes and Obesity
Source: Life (Basel). 2026 Sep 5;16(9):1486. doi: 10.3390/life16091486 (PMC13608023; doi:10.3390/life16091486)
Supplement: Supplementary file 1 [file life-16-01486-s001.zip › life-4446107-supplementary.pdf]

Table S1. Search strategy.

Database	Combination of search terms
PubMed	("glucagon-like peptide 1 receptor agonists"[MeSH Terms] OR "glucagon-like peptide-1 receptor agonist*" OR GLP-1RA OR "GLP-1 receptor agonist*" OR "GLP-1 analog*" OR liraglutide OR semaglutide OR dulaglutide OR exenatide OR lixisenatide OR albiglutide) AND ("pharmacogenetics"[MeSH Terms] OR "genetic variation"[MeSH Terms] OR "single nucleotide polymorphism*" OR SNP OR polymorphism* OR variant* OR mutation* OR allele* OR genotype*) AND ("treatment outcome"[MeSH Terms] OR "drug response" OR "treatment response" OR "lack of response" OR "non-responder*" OR "adverse effect*" OR "side effect*" OR "toxicity" OR "efficacy") AND ("diabetes mellitus, type 2"[MeSH Terms] OR diabet* OR "type 2 diabetes" OR "T2D" OR obesity[MeSH Terms] OR overweight OR "body weight")
Scopus	TITLE-ABS-KEY(“glucagon-like peptide-1 receptor agonist*” OR GLP-1RA OR “GLP-1 analog*” OR liraglutide OR semaglutide OR dulaglutide OR exenatide OR lixisenatide OR albiglutide) AND TITLE-ABS-KEY(“pharmacogenetic*” OR “genetic variant*” OR polymorphism* OR SNP OR mutation* OR genotype* OR allele*) AND TITLE-ABS-KEY(“treatment response” OR “drug response” OR “lack of efficacy” OR “non-responder*” OR “adverse effect*” OR “side effect*” OR toxicity OR efficacy) AND TITLE-ABS-KEY(“type 2 diabetes” OR T2D OR diabetes OR obesity OR overweight)
Web of Science	TS=(“glucagon-like peptide-1 receptor agonist*” OR GLP-1RA OR “GLP-1 analog*” OR liraglutide OR semaglutide OR dulaglutide OR exenatide OR lixisenatide OR albiglutide) AND TS=(“pharmacogenetic*” OR “genetic variant*” OR polymorphism* OR SNP OR mutation* OR genotype* OR allele*) AND TS=(“treatment response” OR “drug response” OR “non-responder*” OR “lack of effect” OR “adverse effect*” OR “side effect*” OR toxicity OR efficacy) AND TS=(“type 2 diabetes” OR T2D OR diabetes OR obesity OR overweight)

Table S2. Baseline characterization of the population in each study.

Author, year	Main morbidities	Mean age (years)	Sex (% female)	Mean BMI (kg/m²)	Glycemic control (HbA1c % and/or FPG mg/dL)	Other treatments (T2DM)
Gavriilidis S, 2024	T2DM (inadequately controlled with metformin ± insulin), hepatic steatosis	64.4 ± 9.5	48.75%	34.5 ± 5.8	HbA1c >7% (inclusion criteria); NR at baseline	Metformin (± basal insulin), SGLT-2 inhibitor, pioglitazone, sulfonylurea
Villikudathil AT, 2024	T2DM	Responders: 56.16 ± 15.25; Non-responders: 60.24 ± 10.42; Controls: 68.37 ± 9.08	Responders: 38.5%; Non-responders: 37.5%; Controls: 50%	NR	HbA1c: Responders 6.4%; Non-responders 8.6%; Controls 5.5%	Other diabetes and non-diabetes medications (concomitant)
Urias E, 2024	Hepatic steatosis / NAFLD (± T2DM, overweight/obesity)	CC: 59.3 ± 11.3; CG/GG: 59.0 ± 10.9	64.1%	36.9 (overall mean)	NR	NR
Kyriakidou A, 2024	T2DM	69.3 ± 10.6	52%	35.8 ± 5.7	HbA1c: Glycemic responders 7.9 ± 1.5%; Non-responders 7.6 ± 0.8%	Other hypoglycemic medications (not specified)
Eghbali M, 2024	T2DM	53.04 ± 8.7	62.7%	31.69 ± 4.8	HbA1c: 9.03 ± 0.91%	Metformin + sulfonylurea or non-sulfonylurea
Dawed AY, 2023	T2DM	54–64 (range of means across cohorts)	47%	Across included studies (NR overall)	HbA1c ≥7% (inclusion criteria)	NR (diverse across cohorts)
Song J, 2022	T2DM (newly diagnosed, drug-naïve)	49.7 (weighted mean)	55%	NR (inclusion: BMI 20–35 kg/m²)	HbA1c 7%–12% (inclusion criteria); NR at baseline	NR (drug-naïve)
Maselli D, 2022	Obesity (BMI >30 kg/m²); otherwise healthy (no T2DM required)	Liraglutide: 42 (32–51); Placebo: 37.2 (29.3–45.2) [median, IQR]	Liraglutide: 88.1%; Placebo: 85.5%	Liraglutide: 35.9 (32.6–40.2); Placebo:	NR	Statins, diuretics, β-blockers, ACE inhibitors/ARBs, metformin (if T2DM/prediabetes)

Author, year	Main morbidities	Mean age (years)	Sex (% female)	Mean BMI (kg/m²)	Glycemic control (HbA1c % and/or FPG mg/dL)	Other treatments (T2DM)
				35.6 (33.1–39.7) [median, IQR]		
Guan Z, 2022	T2DM	50.9 ± 12.7	36.9%	29.4 ± 4.6	HbA1c: 9.1 ± 1.9%	Metformin or other hypoglycemic drugs
Geng Z, 2022	T2DM (newly diagnosed or short-term treatment)	NR (inclusion: 30–70 years)	44%	25.81 ± 2.9	HbA1c: 8.05 ± 1.08%	None (drug-naïve or treatment discontinued)
Kyriakidou A, 2022	T2DM	68.3 ± 10.9	51%	35.4 ± 6.4	HbA1c: Glycemic responders 7.9 ± 1.6%; Non-responders 7.3 ± 0.7%	OHAs (metformin, sulfonylureas, pioglitazone, DPP-4i, SGLT-2i) and/or insulin
Lin C-H, 2021	T2DM (poorly controlled)	Validation group: Responsive 54.6 ± 13.9; Non-responsive 50.7 ± 13.7	Validation: Responsive 47.3%; Non-responsive 58.3%	Validation: Responsive 27.9 ± 5.3; Non-responsive 28.6 ± 5.2	HbA1c: Validation: Responsive 10.4 ± 0.3%; Non-responsive 10.3 ± 0.5%	Other antidiabetic drugs per standard recommendations
Chen Y, 2020	T2DM + NAFLD	I148M+: 51.25 ± 8.73; I148I: 49.17 ± 4.45	I148M+: 25%; I148I: 33%	I148M+: 24.91 ± 2.20; I148I: 25.42 ± 1.19	HbA1c: I148M+: 8.50 ± 0.44%; I148I: 8.68 ± 0.68%	None (monotherapy)
Ferreira MC, 2019	T2DM (<10 years duration)	57.1 ± 7.3	NR	CC: 30.43 ± 4.51; CT/TT: 30.61 ± 5.44	HbA1c: CC: 7.57 ± 1.70%; CT/TT: 7.59 ± 1.78%	NR (insulin, DPP-4i and GLP-1 RA excluded)
Yu M, 2019	T2DM (overweight/obese, inadequate control on metformin)	59.2 ± 10.3	58.9%	36.2 ± 5.0	HbA1c: 9.7 ± 0.95%	Metformin
Chedid V, 2018	Obesity (with accelerated or normal gastric emptying); no T2DM required	Liraglutide: 41.4 ± 2.6; Exenatide: 39.0 ± 3.6; Placebo: 38.4 ± 2.2	Liraglutide: 89%; Exenatide: 60%; Placebo: 80.6%	Liraglutide: 37.4 ± 1.0; Exenatide: 34.6 ± 0.9; Placebo: 35.7 ± 0.7	NR	None (monotherapy vs placebo)

Author, year	Main morbidities	Mean age (years)	Sex (% female)	Mean BMI (kg/m²)	Glycemic control (HbA1c % and/or FPG mg/dL)	Other treatments (T2DM)
Pereira MJ, 2018	Obesity without diabetes (BMI 30–45 kg/m²)	18–70 (range)	60%	NR (inclusion: 30–45 kg/m²)	NR	Dapagliflozin 10 mg once daily
Jensterle M, 2015	PCOS + obesity	27.6 ± 7.2	100%	39.5 ± 6.2	NR	None (prior metformin discontinued)
de Luis DA, 2015	T2DM + overweight/obesity	61.5 ± 9.5	48.9%	33.7 ± 5.9	HbA1c: GG: 8.2 ± 1.1%; GA/AA: 8.6 ± 1.4%	Metformin
de Luis DA, 2014	T2DM + obesity (BMI >30 kg/m²)	60.3 ± 9.6	48.8%	33.2 ± 6.3	HbA1c: 8.6 ± 1.4% (both genotype groups)	Metformin ± sulfonylurea
't Hart LM, 2013	T2DM	NR	NR	NR	NR	NR

* Abbreviations: ACE Inhibitors, Angiotensin-Converting Enzyme Inhibitors; ARBs, angiotensin receptor blockers; BMI, body mass index; DPP-4i, dipeptidyl peptidase-4 inhibitor; FPG, fasting plasma glucose; GLP-1 RA, glucagon-like peptide-1 receptor agonist; HbA1c, glycated hemoglobin; NAFLD, non-alcoholic fatty liver disease; NR, not reported; OHA, oral hypoglycaemic agent; PCOS, polycystic ovary syndrome; SGLT-2i, sodium-glucose cotransporter-2 inhibitor; T2DM, type 2 diabetes mellitus.
